# Ebola Epidemic in Africa as a Global Health Security Threat: A Call for Strengthening Regional Health Diplomacy and ‘One Health’ Implementation

**DOI:** 10.34172/hpp.46207

**Published:** 2026-08-21

**Authors:** Vijay Kumar Chattu, Hamid Allahverdipour

**Affiliations:** ^1^ReSTORE lab, Department of OS & OT, Temerty Faculty of Medicine, University of Toronto, Canada; ^2^Central Research Facility, Dr. D.Y Patil Medical College, Hospital and Research Centre, Dr. D.Y Patil Vidyapeeth, Pune, India; ^3^United Nations University Institute on Comparative Regional Integration Studies (UNU-CRIS), Potterierei, Bruges, Belgium; ^4^Advisory Board, Health Promotion Perspectives; ^5^Editor-in-Chief, Health Promotion Perspectives; ^6^Department of Health Education & Promotion, Tabriz University of Medical Sciences, Tabriz, Iran; ^7^Research Center of Psychiatry and Behavioral Sciences, Tabriz University of Medical Sciences, Tabriz, Iran

“*The One Health approach is no longer a choice. It is a strategic imperative in an unstable but increasingly interconnected world,”- Dr. Jeremy Farrar, Assistant Director-General, WHO*

## Introduction

 The World Health Organization (WHO) has recognized the Ebola epidemic in the Democratic Republic of the Congo (DRC) and Uganda as a public health emergency of international concern (PHEIC), marking the first such announcement in 2026. Still, it doesn’t meet the pandemic emergency criteria as per the International Health Regulations (2005) (IHR).^1^ The outbreak is caused by the Bundibugyo virus strain, an Ebola virus variant for which there is no recognized vaccine or treatment.^2^ Fears regarding the magnitude of the current Ebola outbreak in the Democratic Republic of the Congo are being raised by an absence of vaccines, diagnostics/ therapeutics, and basic public health capabilities. The epidemic remains a critical threat to global health security, and due to the modern transport and global travel infrastructure, the disease can easily spread from villages to international hubs within a short time.

## What is Ebola virus Disease?

 Ebola virus disease is a severe, often fatal illness that was first transmitted to humans by wild animals (such as fruit bats, porcupines, and non-human primates) and then spread from person to person through direct contact with an infected person’s blood, secretions, organs, or other bodily fluids (Figure 1). It can also be transmitted through fluid-contaminated surfaces and materials (for example, bedding and clothing).^3^ The virus was co-discovered by Profs Jean-Jaques Muyembe, Guido van der Groen, and Peter Piot in 1976 in Congo’s Ebola River region.^4^

**Figure 1:**
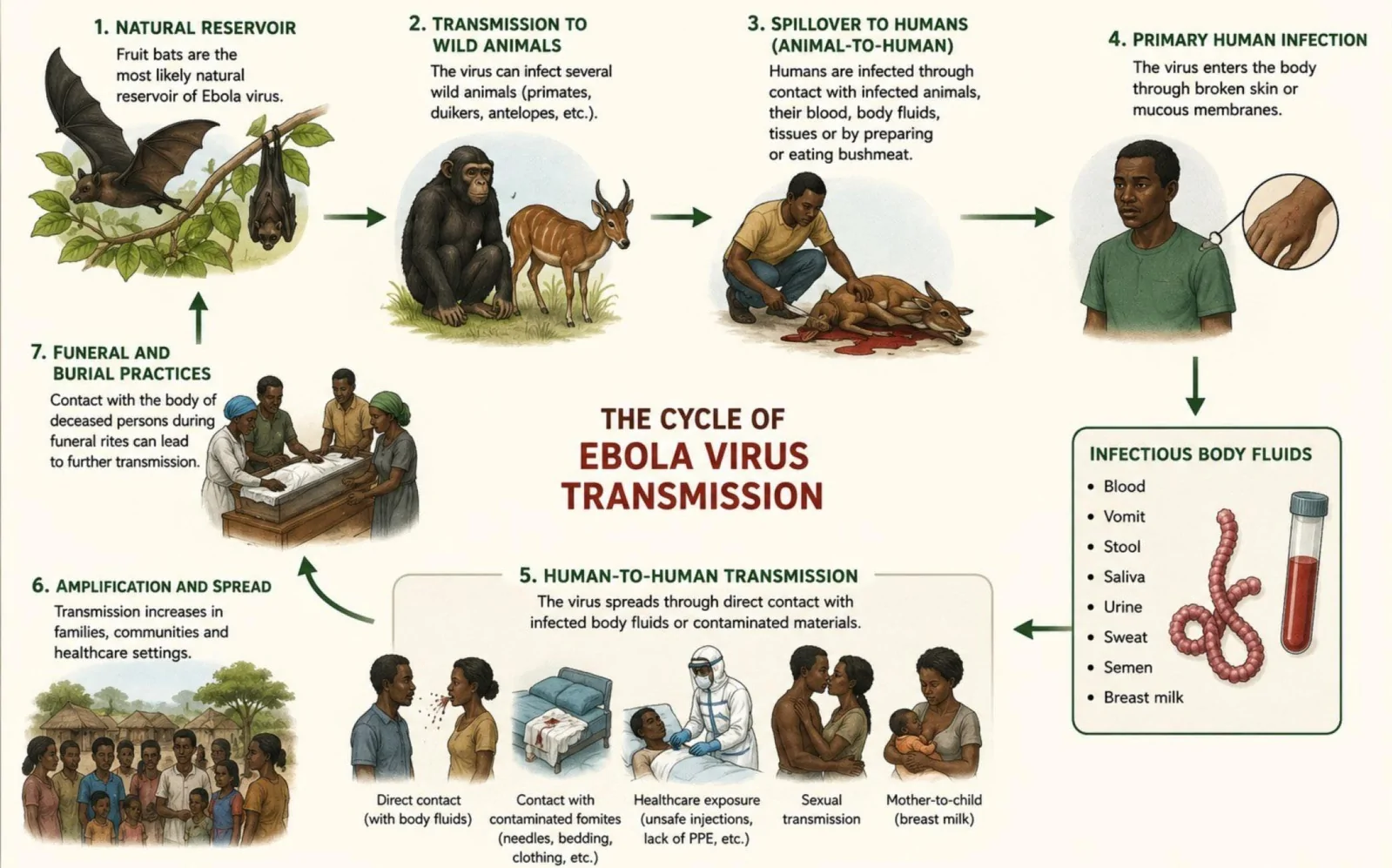
Schematic representation of the cycle of Ebola virus transmission (Figure developed using ChatGPT with the prompt to “show the cycle of Ebola virus transmission”) As shown in Table 1, there have been numerous Ebola outbreaks since 1976 caused by various ebolavirus species, with the major epidemic that happened in West Africa in 2014 resulting in over 11,308 deaths.^5^ The DRC has the most frequent epidemics caused by Orthoebolavirus *zairensis, *and thecase fatality rate in these epidemics ranges from 36% to 100%.

 As shown in Table 1, there have been numerous Ebola outbreaks since 1976 caused by various ebolavirus species, with the major epidemic that happened in West Africa in 2014 resulting in over 11,308 deaths.^5^ The DRC has the most frequent epidemics caused by Orthoebolavirus *zairensis, *and thecase fatality rate in these epidemics ranges from 36% to 100%.

**Table 1:** History of Ebola Outbreaks from 1976 to date (15 July 2026)

**S No**	**Year**	**Country**	**Genus Species**	**Infections**	**Deaths (Case Fatality Rate)**
1	2026	DRC Uganda France Germany	Orthoebolavirus *bundibugyoense* Orthoebolavirus *bundibugyoense* Orthoebolavirus *bundibugyoense* Orthoebolavirus *bundibugyoense*	2124 20 1 1	828 (39%) 2 (10%)
2	2025	DRC	Orthoebolavirus *zairense*	64	45 (70%)
3	2022	Uganda DRC DRC	Orthoebolavirus *sudanense* Orthoebolavirus *zairense* Orthoebolavirus *zairense*	164 1 5	55 (34%) 1 (100%) 5 (100%)
4	2021	DRC	Orthoebolavirus *zairense*	11	9 (82%)
5	2020	DRC	Orthoebolavirus *zairense*	130	55 (42%)
6	2018	DRC DRC	Orthoebolavirus *zairense* Orthoebolavirus *zairense*	3,470 54	2,287 (66%) 33 (61%)
7	2017	DRC	Orthoebolavirus *zairense*	8	4 (50%)
8	2014-2016	DRC Guinea, Liberia, Sierra Leone Italy Mali Nigeria Senegal Spain UK US	Orthoebolavirus *zairense* Orthoebolavirus *zairense* Orthoebolavirus *zairense* Orthoebolavirus *zairense* Orthoebolavirus *zairense* Orthoebolavirus *zairense* Orthoebolavirus *zairense* Orthoebolavirus *zairense* Orthoebolavirus *zairense* Orthoebolavirus *zairense* Orthoebolavirus *zairense*	69 3811 10675 14124 1 8 20 1 1 1 4	49 (71%) 2543 (67%) 4809 (45%) 3956 (28%) 0 6 (75%) 8 (40%) 0 0 0 1 (25%)
9	2012	Uganda DRC Uganda	Orthoebolavirus *sudanense* Orthoebolavirus *bundibugyoense* Orthoebolavirus *sudanense*	6 62 11	3 (50%) 34 (55%) 4 (36%)
10	2011	Uganda	Orthoebolavirus *sudanense*	1	1 (100%)
11	2008	DRC Philippines	Orthoebolavirus *zairense* Orthoebolavirus *restonense*	32 6	15 (47%) 0
12	2007	Uganda DRC	Orthoebolavirus *bundibugyoense* Orthoebolavirus *zairense*	131 264	42 (32%) 187(71%)
13	2005	Republic of Congo	Orthoebolavirus *zairense*	12	10 (83%)
14	2004	Russia Sudan	Orthoebolavirus *zairense* Orthoebolavirus *sudanense*	1 17	1 (100%) 7 (41%)
15	2003	Republic of Congo Republic of Congo	Orthoebolavirus *zairense* Orthoebolavirus *zairense*	35 143	29 (83%) 128 (89%)
16	2001	DRC	Orthoebolavirus *zairense*	59	44 (75%)
17	2000	Uganda	Orthoebolavirus *sudanense*	425	224 (53%)
18	1996	Russia Philippines United States South Africa Gabon Gabon	Orthoebolavirus *zairense* Orthoebolavirus *restonense* Orthoebolavirus *restonense* Orthoebolavirus *zairense* Orthoebolavirus *zairense* Orthoebolavirus *zairense*	1 0 0 2 60 31	1 (100%) 0 0 1 (50%) 45 (75%) 21 (68%)
19	1995	DRC	Orthoebolavirus *zairense*	315	254 (81%)
20	1994	Cote d’Ivoire Gabon	Orthoebolavirus *taiense* Orthoebolavirus *zairense*	1 51	0 31
21	1992	Italy*	Orthoebolavirus *restonense*	0	0
22	1989	Philippines	Orthoebolavirus *restonense*	3	0
23	1979	Sudan	Orthoebolavirus *sudanense*	34	22 (65%)
24	1977	DRC	Orthoebolavirus *zairense*	1	1 (100%)
25	1976	UK Sudan DRC	Orthoebolavirus *zairense* Orthoebolavirus *sudanense* Orthoebolavirus *zairense*	1 284 318	0 151 (53%) 280 (88%)

Prepared by the authors. (**source**: Africa CDC^3^ and CDC Atlanta^6^) * *Reston virus was introduced into quarantine facilities in Sienna by monkeys imported from the same export facility in the Philippines that was involved in the episodes in the United States. People were not infected.*

## Critical Role of Regional Health Diplomacy

 Regional challenges encompass disease outbreaks/ epidemics, the rising burden of NCDs, war, displacement, climate migration, instability, and insecurity stemming from geopolitical issues.^7^ Regional Health Diplomacy engages diverse stakeholders, such as ministerial departments in public health, international law, international affairs, economics, Information Technology, and management, to strengthen cooperation, shape the regional health policy environment, and promote peace in the region through successful negotiations.^8^ Countries need to prioritize and engage in RHD because they are disproportionately affected by man-made and humanitarian disasters, and many of the development challenges they face are directly tied to health. There is a great need to pool resources, share technologies, and implement effective strategies to address health threats and challenges, such as strengthening and implementing ‘One Health’ to handle the Ebola epidemic. Besides, promoting “Health in All Policies” should be critical for all countries to reduce health disparities and strengthen health systems. RHD helps to achieve global goals by strengthening relationships between states and promoting regional development and effective governance.Additionally, it was emphasized that successful RHD might foster global solidarity, multilateralism, international cooperation, and efficient regional governance systems to prevent infectious diseases and NCDs.^9^

 Thus, RHD, as a health promotion approach, is crucial for improving regional contact and cooperation across health, foreign policy, and other domains. Improving health, along with education, and addressing equity gaps are critical for both national security and regional socioeconomic development, and nations/regions should prioritize them amid a weakening multilateral system, geopolitical tensions, and the widespread adoption of more nationalist agendas.^10^

## One Health Strategy and its relevance for the Ebola epidemic:

 One Health Strategy is a comprehensive, multisectoral framework for harmonizing and balancing the health of humans, animals, and ecosystems. Health, food, water, energy, and the environment are all broader issues with sector-specific concerns. The integrity and health of our ecosystems are supported, and health issues such as rising infectious diseases, antimicrobial resistance (AMR), and food safety are addressed through cross-sector and cross-disciplinary collaboration.^11^ Ebola is a zoonotic virus that originates in wildlife reservoirs such as fruit bats and apes; thus, tackling it only in humans is inadequate. One Health, by connecting humans, animals, and the environment, can assist in addressing the entire spectrum of Ebola disease control - from prevention to detection, preparedness, response, and management - while also promoting global health security.^11^ For example, even in the case of the past Nipah virus outbreak, it was emphasized that ‘One Health’ approach ensures global health security as it brings in the multi-disciplinary, multi-sectoral expertise that addresses the human health, animal health, and the environment.^12^

 Recently, during the One Health Summit in Lyon on 7 April 2026, the Quadripartite collaboration, comprising the Food and Agriculture Organization of the United Nations (FAO), the United Nations Environment Programme (UNEP), the World Health Organization (WHO), and the World Organisation for Animal Health (WOAH), united with global leaders and partners to advocate for enhanced investment and collaboration, as well as expedited implementation of the One Health approach at the national level.^13^ The strategy can be used at the community, subnational, national, regional, and global levels and is based on shared and effective governance, communication, collaboration, and coordination. Having the One Health strategy in place enables individuals to better understand the co-benefits, risks, trade-offs, and opportunities for advancing equitable and comprehensive solutions.^11^ In the context of Central Asia, Chattu et al have highlighted that RHD is the most effective way to achieve One Health as a cross-sectoral strategy, offering a solid foundation for negotiating shared priorities and averting future pandemics.^14^

## Strengthening African Regional Cooperation

 Regional cooperation is critical for the prevention and control of outbreaks and epidemics through community engagement. Delivering appropriate services and a range of interventions to the affected populations in the region, raising awareness of risk factors, providing clinical care, protective measures, surveillance, infection prevention and control, etc., needs effective coordination. To strengthen regional cooperation, the following activities are vital.^1^

###  High-level political prioritization

 It is vital to ensure high-level political commitment for effective coordination and communication, and to address all circulating misinformation and disinformation. Ashighlighted by the WHO,initiate national disaster and emergency management protocols and establish an emergency operations center under the Head of State’s jurisdiction, with appropriate governmental authority.

###  Coordination of Response activities 

 Coordination and response systems must be established at the regional, national, and subnational levels in affected and at-risk regions to contain the infection by implementing evidence-based strategies and guidelines. Cooperation between national governments, the private sector, business entities, and civil society at the regional, national, and local levels is necessary for a robust and sustainable plan to combat the ongoing Ebola outbreak. Engaging in RHD can help to build political and community support, coordinate responses, improve access to care, and create opportunities for community and political engagement.^7^

###  Risk communication and community engagement (RCCE)

 RCCE is the fundamental strategy for facilitating a comprehensive, ongoing initiative to actively engage the community—using local, religious, and traditional leaders and healers—so that communities play an important role in case identification, contact tracing, and risk education; the public must be fully informed about the benefits of early treatment. Trust is essential for disease prevention, especially in areas where many people are fearful. As highlighted in the recent Lancet Ebola special issue, there is a sense of fear in the community and many uncertainties, worsened by the ongoing conflict. Thus, it is critical to ensure that local workers are well-trained and clearly understand what they are dealing with so they can mobilize others.^15^ Therefore, efforts should focus on improving community understanding, engagement, and participation by identifying and challenging cultural norms and attitudes that impede full participation in the response. Furthermore, incorporate the reaction into the overall strategy for meeting the population’s needs.

###  Surveillance and Resource sharing

 Provide medical experts, diagnostic tools, and fast response teams to the most vulnerable and conflict-affected communities.

####  Strengthening Surveillance and Laboratory Capacity:

 Strengthening surveillance and laboratory capacities in affected and neighboring provinces through the establishment of (1) specialized surveillance and response units in impacted health zones and critical at-risk adjacent areas, (2) enhanced community surveillance, with a focus on community fatalities, and (3) decentralized laboratory facilities for virus testing.^16^

###  Prevention and Control and Treatment Activities

####  Infection prevention and control (IPC) activities:

 Enhance strategies to prevent nosocomial infections, including thorough mapping of healthcare facilities, triage, targeted infection prevention and control actions, and continuous monitoring and oversight. Ensure that healthcare personnel receive comprehensive IPC training, including proper use of PPE, and that health facilities have the necessary equipment to protect staff, ensure prompt salary payments, and, if appropriate, provide hazard pay.

####  Controlling infection in healthcare centers and hospitals

 Policies must be in place for all healthcare personnel caring for those with suspected or confirmed cases.

####  Preventing Economic Panic

 Establish uniform regional travel and trade regulations to prevent substantial economic turmoil caused by uncoordinated border shutdowns.

####  Treatment and Vaccination Services

 The WHO strongly advised using monoclonal antibodies, either REGN-EB3 (InmazebTM) or mAb114 (ansuvimabTM), to treat Ebola virus sickness. There are no licensed therapeutics for additional Ebola disorders like SVD or BVD, but potential treatments are being developed. Ervebo (Merck & Co.) and Zabdeno Mvabea (Janssen Pharmaceutica) are the two approved vaccines. As part of the outbreak response, the Ervebo vaccine is advised, and numerous vaccine candidates are in various stages of development.^16^

###  Care for survivors

####  Ensure a continuum of care for suspected and infected cases:

 Through a patient-centered and humanistic approach, ensure that suspected cases are referred and safely transferred to specialized clinical facilities for isolation and management. Dedicated treatment facilities should be established in close proximity to the outbreak epicenter(s). These facilities should be staffed by personnel who are trained and prepared to provide the best possible intensive supportive care.

####  Tracking Border health, travel, and mass-gathering events: 

 To contain the international spread of the virus, conduct cross-border screening and screening at major internal highways to ensure that no suspected cases are overlooked, and enhance screening quality by more effectively exchanging information with surveillance teams. Unless it is part of an approved medical evacuation, international travel for Bundibugyo virus disease contacts or cases should be avoided. To mitigate the risk of international transmission of Bundibugyo virus disease.

## Regional Health Governance during the crisis

 Regional Health Governance aims to promote collaboration and enhance health outcomes within a particular region. To address common health issues and advance regional stability, it brings together a variety of actors, including governments, international organizations, and civil society. This strategy acknowledges that regional collaboration can yield more efficient and sustainable solutions and that health is not just a national problem.^17^ Efforts should focus on the effective implementation of the International Health Regulations 2005 (IHRs), strengthening the health systems, and securing financial resources from various stakeholders through negotiations. Besides, to improve communication and cooperation across foreign policy, health, and other areas at the regional level, RHD is essential as a health-promotion approach. Chattu et al, in a recent article, highlighted the role of RHD in the Central Asian collaboration in One Health, representing a paradigm shift away from fragmented state health initiatives toward an integrated regional approach that leverages shared geopolitical, historical, and epidemiological vulnerabilities to build collective resilience.^14^ Similarly, in Africa, there must be ongoing cooperation and communication between the regional partners in health- Africa CDC, national health systems, research organizations, business organizations, the pharmaceutical industry, the African Medicines Agency, UN agencies, and other professional associations with a common goal to strengthen global health security.

## Conclusion

 The current Bundibugyo Ebola virus epidemic remains a critical threat to global health security, causing many deaths in the Democratic Republic of Congo, and is a source of fear in the community, with many uncertainties, worsened by the ongoing conflict. The African region must ensure high-level political commitment for effective coordination and communication to address the epidemic before it crosses borders. RHD helps achieve goals by strengthening relationships between states and promoting regional development and effective governance; successful RHD might foster global solidarity and multilateralism. ‘One Health’ strategy ensures global health security as it brings in the multi-disciplinary, multi-sectoral expertise that addresses the human health, animal health, and the environment. We conclude that RHD is the most effective way to achieve One Health as a cross-sectoral strategy, offering a solid foundation for negotiating shared priorities and averting future pandemics.

## Ethical approval

 Not Applicable

## Competing Interests

 Vijay Kumar Chattu is an Editorial Board Member, and Hamid Allahverdipour is the Editor-in-Chief of Health Promotion Perspectives.
